# On-Chip Asymmetric Microsupercapacitors Combining Reduced Graphene Oxide and Manganese Oxide for High Energy-Power Tradeoff

**DOI:** 10.3390/mi9080399

**Published:** 2018-08-12

**Authors:** Richa Agrawal, Chunlei Wang

**Affiliations:** 1 Department of Mechanical and Materials Engineering, Florida International University, Miami, FL 33174, USA; ragra005@fiu.edu; 2 Center for the Study of Matter of Extreme Conditions (CeSMEC), Florida International University, Miami, FL 33199, USA

**Keywords:** asymmetric electrochemical capacitors, interdigitated microsupercapacitors, electrophoretic deposition, reduced graphene oxide, manganese oxide

## Abstract

Given the rapid miniaturization of technology, it is of interest to produce viable on-chip micro-electrochemical energy storage systems. In this study, interdigitated asymmetric microsupercapacitors were fabricated using photolithography, lift-off and electrodeposition methods. Manganese oxide (MnO_x_) and reduced graphene oxide (rGO) comprised the pseudocapacitive and the double layer component, respectively. Symmetric MnO_x_//MnO_x_, rGO//rGO as well as asymmetric rGO//MnO_x_ microsupercapacitors with three different MnO_x_ thicknesses were constructed and characterized in aqueous media. The asymmetric microsupercapacitor with the intermediate MnO_x_ film thickness displayed the optimal energy-power trade-off superior to that of both the symmetric and well as the other asymmetric configurations. The optimal microsupercapacitor exhibited a high stack energy density of 1.02 mWh·cm^−3^ and a maximal power density of 3.44 W·cm^−3^. The high energy-power trade-off of the device is attributed to the synergistic effects of utilizing double layer and pseudocapacitive charge storage mechanisms along with in-plane interdigital microelectrode design within one optimized micro-device.

## 1. Introduction

With the technological impetus of going “micro”, it is imperative to create small-scale energy devices that can effectively power such miniaturized devices. Given the existing myriad of minuscule systems such as implantable medical devices (IMDs), wireless sensors, smart cards, personal electronics, etc. that typically require power in the range of several µW to several hundred mW, it is essential to shrink the sizes of the energy storage components further. Currently, the majority of the micro-devices rely on batteries to provide the required energy and power. However the relatively poor power-handling ability and limited lifetime of batteries hinder their applicability to systems that require high current spikes [1]. As an alternative to batteries, energy harvesters hold significant promise for sustainable environments; however, the currently existing energy harvester systems require an energy storage device in tandem [1]. Electrochemical capacitors, also known as supercapacitors (SCs), on the other hand, can provide high powers along with long cycle lives. Based on the charge storage mechanisms, SCs can be divided into two major categories—electrochemical double layer capacitors (EDLCs) and redox or “pseudocapacitors”. The former rely on the adsorption of ions at the electrode/electrolyte interface and typically comprise different forms of carbons, whereas the latter store charges in a faradaic/redox manner with fast and reversible redox reactions and encompass different transition metal oxides (TMOs) and conjugated polymers [2]. Typically, pseudocapacitive materials exhibit larger specific capacitances, whereas double layer type materials exhibit better rate handling capability and superior cycle longevity [2].

Quite akin to their larger variants, miniaturized electrochemical energy storage (EES) systems can be connected externally for peak power and energy delivery; nevertheless, it is desirable for the next generation micro-power devices to be single standalone systems that can provide with simultaneous supply of high energy and high power. One of the strategies for achieving the latter is the asymmetric or hybrid supercapacitor design. Such systems typically combine a redox-type electrode along with a counter double layer capacitive electrode in one cohesive system and benefit from the larger capacity of the redox material and the superior kinetics and cycle life of the double layer material. Several asymmetric systems have been reported in the literature including pseudocapacitive metal oxides such as ruthenium oxide (RuO_2_) [3], manganese oxide (MnO_x_) [4,5,6,7,8], nickel oxide (NiO_x_) [9,10], as well as lithium insertion materials (lithium-ion capacitors) including pre-lithiated carbons [11,12,13], lithium titanate (Li_4_Ti_5_O_12_) [14,15,16,17], and lithium iron phosphate (LiFePO_4_) [18,19] coupled with different carbons. While the asymmetric design offers significant promise from materials perspective, the “in-plane interdigital design” offers a multitude of advantages from an architectural standpoint. For instance, having alternating digits of anode and cathode materials in close proximity can shorten the ion-transport pathways and can effectively enhance the rate capability of the on-chip systems. As opposed to the conventional two-dimensional (2D) thin-film design, the interdigital architecture allows for larger accessibility of the electrodes as the sides of the microelectrodes are exposed to the electrolyte [1]. The enhanced accessibility of the electrodes is especially well-suited for layered 2D materials, where the electrolyte ions can have facile access between the layers of the material by having the electrodes side-by-side [1].

One of the widely investigated 2D layered materials is graphene, which is essentially a one-atom thick sp^2^ hybridized carbon sheet [2]. Owing to its high theoretical specific surface area (~2600 m^2^/g), exceptional electronic conductivity, and superior mechanical strength, graphene has been extensively explored as an EDLC material [2,20,21,22]. Despite the phenomenal properties of graphene, it is, however, challenging to fully realize the theoretical values, given the high degree of graphene sheet restacking [2,12]. Furthermore, the hydrophobic nature of graphene makes it challenging to effectively disperse it in solvents. One of the derivatives of graphene is graphene oxide (GO), which in essence is graphene decorated with oxygen-containing groups on both the basal planes and the edges [23,24]. The oxygen functionality makes GO hydrophilic, which can assist in its effective dispersion in solvents as well as enhance the interaction with the electrolyte [25]. However, the oxygenated groups make GO insulating and for SC applications, effective reduction of GO is desired in order to utilize the electronic properties of graphene. Typically, GO is reduced to reduced graphene oxide (rGO) using strong reducing agents like hydrazine, which could be both cost-prohibitive and damaging to the environment [25]. As an alternative to chemical reduction, electrophoretic deposition (EPD) offers the possibility to effectively reduce GO without the need for any additional reducing agents [26]; An et al. [27] noted that the rGO films synthesized via EPD exhibited much superior electrical conductivity over the parent GO papers (1.43 × 10^4^ S·m^−1^ as opposed to 0.53 × 10^−3^ S·m^−1^). EPD is a thin-film synthesis method, which essentially involves the movement of charged particles in a colloidal suspension under the influence of an electric field [26,28]. Given the feasibility to simultaneously integrate and reduce GO using EPD, the latter method was utilized to fabricate the rGO microelectrodes for the microsupercapacitors (MSCs) for this work.

As pseudocapacitive or redox materials, manganese oxides (MnO_x_), in all polymorphs, have been widely studied owing to their high theoretical specific capacitance, environmental benignity, large abundance and low cost [29,30,31,32]. Different asymmetric configurations utilizing MnO_x_ have been investigated for MSC applications including activated carbons (AC) [33], carbon nanotubes (CNT) [34], laser scribed graphene (LSG) [35], as well as graphene quantum dot (GQD) [36] counter electrodes. In this work, MnO_x_ was electrochemically deposited onto gold micro-current collectors as the cathode component of the asymmetric MSCs against the electrophoretically prepared rGO counter microelectrode. The optimized asymmetric rGO//MnO_x_ MSC was able to deliver areal capacitances as high as 1.63 mF·cm^−2^, equivalent to a stack/volumetric capacitance of 3.6 F·cm^−3^ as well as volumetric energy and power densities of 1.02 mWh·cm^−3^ and 3441 mW·cm^−3^, much superior to those of both the symmetric MnO_x_//MnO_x_ and rGO//rGO systems. The excellent energy-power tradeoff and high specific capacitance of the asymmetric capacitor is attributed to the synergy between the pseudocapacitive MnO_x_ component and the double layer rGO component in addition to the in-plane interdigital microelectrode design.

## 2. Materials and Methods

### 2.1. Fabrication of the Interdigital Gold Micro-Current Collectors

**Figure 1:**
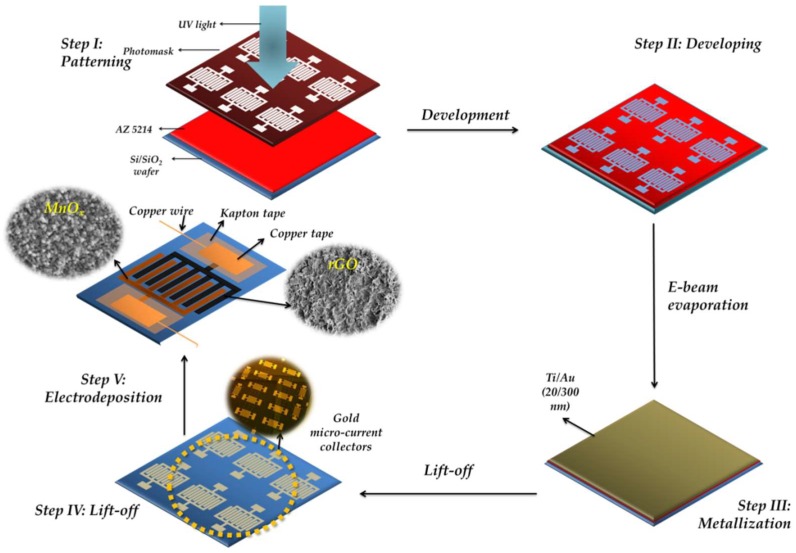
Schematic illustration of the asymmetric rGO//MnO_x_ microsupercapacitor construction.

To construct the interdigital gold micro-current collectors, conventional photolithography and lift-off methods were used. A schematic illustration has been shown in Figure 1 (*Step I-Step IV*). First, AZ 5214 (Microchemicals, Ulm, Germany), a positive-tone photoresist, was spun-coated on a 4” Si/SiO_2_ (100) wafer at a speed of 5000 rpm for 30 s. After the spin coating process, a soft bake was carried out for 1 min at a temperature of 110 °C on a leveled hot plate. After the bake process, ultraviolet exposure was carried out using an OAI mask aligner with a UV dose of 230 mJ·cm^−2^. Following the expose step, a develop step was carried out using AZ 300 MIF (Integrated Micro Materials, Argyle, TX, USA) for ~45 s. After the develop step, a de-scum step was carried out using an O_2_ plasma at a flow rate of 60 sccm and a pressure of 400 mTorr for 30 s using reactive ion etching (RIE). Following the patterning of the photoresist, metallization process (Ti/Au (20/300 nm)) was carried out using e-beam evaporation with a CHA evaporator (CHA Industries, Inc., Fremont, CA, USA). After the metallization process, lift-off was carried out in order to get rid of sacrificial photoresist using acetone and mechanical agitation, followed with isopropyl alcohol rinsing and N_2_ drying. Finally, a 30 s O_2_ plasma treatment at 400 mTorr was carried out in order to get rid of any organic residues. The typical width of the gold fingers was 100 µm with an interdigital gap of 100 µm and a length of 8800 μm. Each electrode comprised 18 fingers, resulting in a total of 36 fingers for a single device and a total finger area of 0.3168 cm^2^, whereas the effective footprint area of the device (including finger gaps) was ~0.66 cm^2^. Unless otherwise mentioned, the electrochemical parameters were normalized with the finger area in this work.

### 2.2. Active Material Integration

After constructing the gold micro-current collectors, the active materials (rGO and MnO_x_) were integrated onto the current collectors using electrodeposition (electrochemical and electrophoretic deposition methods). For the symmetric MnO_x_//MnO_x_ microsupercapacitor, a three-electrode setup was used where the interdigitated gold current collectors functioned as the working electrode, and an Ag/AgCl electrode (and a platinum coil functioned as the reference and counter electrode, respectively). An anodic current of 0.5 mA·cm^−2^ was applied for 20 min (equivalent to an applied charge of 0.6 C·cm^−2^) in an electrolyte solution containing 0.2M manganese acetate (Sigma-Aldrich, St. Louis, MO, USA) and 0.2 M Na_2_SO_4_ (Sigma-Aldrich, St. Louis, MO, USA). After the deposition process, the electrodes were washed with deionized (DI) water and dried overnight before being used for further characterization. For the rGO deposition for the symmetric rGO//rGO microsupercapacitor, a suspension containing single layer graphene oxide (SLGO, Cheap Tubes Inc., Cambridgeport, VT, USA) and C_2_H_5_OH:DI water (90:10, v:v) in a concentration of 1 mg·mL^−1^ was used for the EPD process after 1 h ultrasonication. An electric field of ~40 V cm^−1^ was applied between the gold current collectors (working electrode) and a platinum coil (counter electrode); small amounts of MgCl_2_ (0.1 mg·mL^−1^) were added to the GO solution in order to enhance the conductivity and facilitate the EPD process. For the asymmetric microsupercapacitors (schematic illustration shown in Figure 1 Step *V*), three different depositions of manganese oxide comprising anodic charges of 0.6, 0.9 and 1.2 C·cm^−2^ were evaluated against a 5 min deposition of rGO. rGO was deposited first on one of the interdigital electrodes as described earlier, followed by the respective MnO_x_ deposition on the counter electrode. The device was washed several times with DI water and dried thoroughly prior to its characterization.

### 2.3. Material Characterization

X-ray diffraction (XRD) studies on the electrochemically deposited MnO_x_ were carried out using a Siemens D5000 Diffractometer (Siemens, Munich, Germany) with Cu-Kα radiation. For spectroscopic characterization on the electrochemically deposited MnO_x_ and the electrophoretically rGO films, Fourier Transform Infrared (FTIR) studies were carried out using a JASCO FTIR-4100 (JASCO, Easton, MD, USA) equipped with an attenuated total reflectance (ATR) accessory The top and the cross sectional views of the symmetric and asymmetric microsupercapacitors were investigated using scanning electron microscopy (SEM) with a JEOL SEM 6330 (JEOL, Peabody, MA, USA) in the secondary electron imaging (SEI) mode.

### 2.4. Electrochemical Characterization

The electrochemical characterization on the symmetric rGO//rGO, MnO_x_//MnO_x_, as well as the asymmetric rGO//MnO_x_ microsupercapacitors was carried out in an aqueous electrolyte containing 1 M Na_2_SO_4_ using a Bio-logic Versatile Multichannel Potentiostat (VMP3) (Bio-Logic, Seyssinet-Pariset, France). Two-electrode studies were carried out for both the symmetric and asymmetric configurations and all the experiments were carried out at room temperature, in a sealed beaker-type cell assembly. All the electrochemical parameters were normalized with the total finger/active material area and the systems were evaluated using cyclic voltammetry (CV), galvanostatic charging and discharging (GCD), as well as electrochemical impedance spectroscopy (EIS) measurements.

## 3. Results

### 3.1. Spectroscopic, Crystallographic and Microstructural Characterization Performed on the Manganese Oxide and rGO Microelectrodes

**Figure 2:**
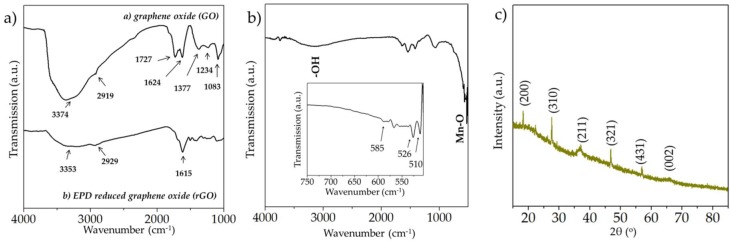
(**a**) FTIR spectra of the starting graphene oxide powders and the EPD reduced graphene oxide films; (**b**) FTIR spectrum and (**c**) the X-ray diffraction (XRD) pattern of the electrochemically deposited MnO_x_ films.

The FTIR spectra of the starting GO powders and the EPD reduced GO (rGO) is shown in Figure 2a. The broad absorption peak centered around 3374 cm^−1^ in the GO powder is the characteristic infrared (IR) band position from the OH stretching vibrations [37,38], whereas the peaks at 1727, 1624, 1377, 1234, and 1083 cm^−1^ are attributed to C=O stretching [38], aromatic C=C stretching [39], carboxyl [40], epoxide C-O-C or phenolic C-O-H stretching vibrations [40], C-O stretching in epoxy or alkoxy groups [40], respectively. It is worth noting that the intensity from the hydroxyl groups is substantially mitigated in the EPD-based rGO film, indicating effective reduction of GO during electrophoresis. Furthermore, the intensity of the other functional groups signaling the presence of oxygen was reduced; the peak at 1615 cm^−1^ was however quite prevalent in the rGO spectrum, signaling the presence of aromatic C=C stretching [39], which is from the parental graphitic skeleton.

The FTIR spectrum of the electrodeposited MnO_x_ is shown in Figure 2b; the peaks located around 510, 526, and 585 cm^−1^ (below 750 cm^−1^, shown as the inset) are ascribed to the Mn-O vibrations from the MnO_6_ octahedra and are consistent with previous reports [41,42]. The XRD pattern of the electrochemically deposited manganese oxide films is shown in Figure 2c. The material is of low degree of crystallinity; however the faint peaks present in the diffraction pattern are ascribed to α-MnO_2_ (JCPDS Card Number: 00-044-0141). The peaks at 18.1°, 28.8°, 37.5°, 46.1°, 56.9°, and 65.2° are indexed as (hkl) plane orientations of (200), (310), (211), (321), (431), and (002), respectively of α-MnO_2_ phase. However, owing to the poor degree of crystallinity the manganese oxide is referred to as MnO_x_.

**Figure 3:**
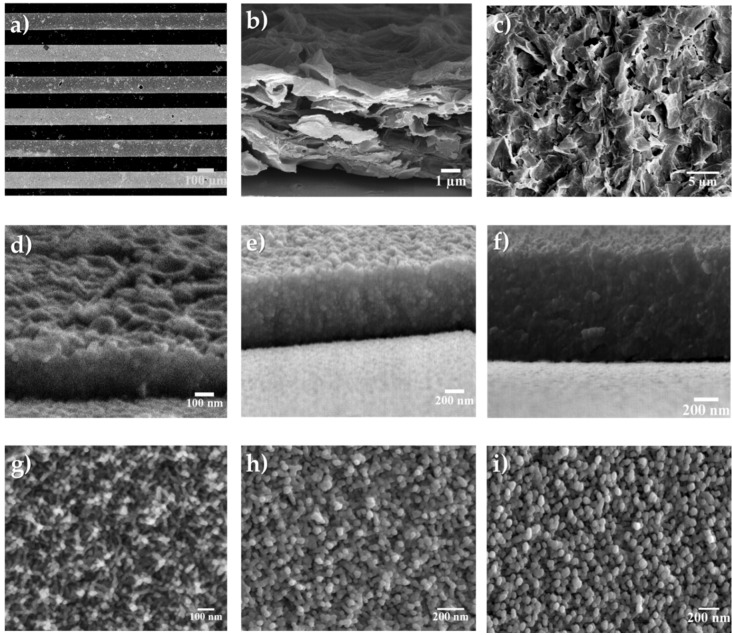
(**a**) SEM micrograph of the interdigitated rGO and MnO_x_ microelectrodes; (**b**) cross sectional view and (**c**) top view of the rGO coated microelectrodes; (**d**–**f**) cross sectional view of the MnO_x_ microelectrodes deposited at 0.6, 0.9 and 1.2 C·cm^−2^, respectively; (**g**–**i**) top-view of the MnO_x_ microelectrodes deposited at 0.6, 0.9 and 1.2 C·cm^−2^, respectively.

The top view of the asymmetric interdigitated MSC is shown in Figure 3a. Figure 3b displays the cross-sectional view of the rGO electrode—the structure consisted of graphene layers as expected from the parent graphene oxide powders (SEM micrograph shown in Supplementary file Figure S1) and the rGO film had an average thickness of ~4.5 μm. The top view of the rGO microelectrode is illustrated in Figure 3c; as evident the graphene sheets were well dispersed, thereby confirming effective sheet assembly during the EPD process. Figure 3d–f show the cross sectional images of the MnO_x_ films deposited at anodic charges of 0.6, 0.9 and 1.2 C·cm^−2^ and the corresponding morphologies of the films are exhibited as Figure 3g through Figure 3i, respectively. As expected, the MnO_x_ film deposited for an anodic charge of 0.6 C·cm^−2^ was the thinnest among the films deposited for different charges. The average film thicknesses for the MnO_x_ microelectrodes were ~0.35, ~0.81, and ~0.95 μm (tilt adjusted) for deposition rates of 0.6, 0.9 and 1.2 C·cm^−2^, respectively. The microstructure of the different MnO_x_ films was similar and comprised homogeneously dispersed MnO_x_ nanoparticles.

### 3.2. Electrochemical Characterization of the Symmetric rGO//rGO, MnO_x_//MnO_x_, and Asymmetric rGO-MnO_x_ Microsupercapacitors

#### 3.2.1. Electrochemical Evaluation of the Symmetric rGO//rGO MSCs

**Figure 4:**
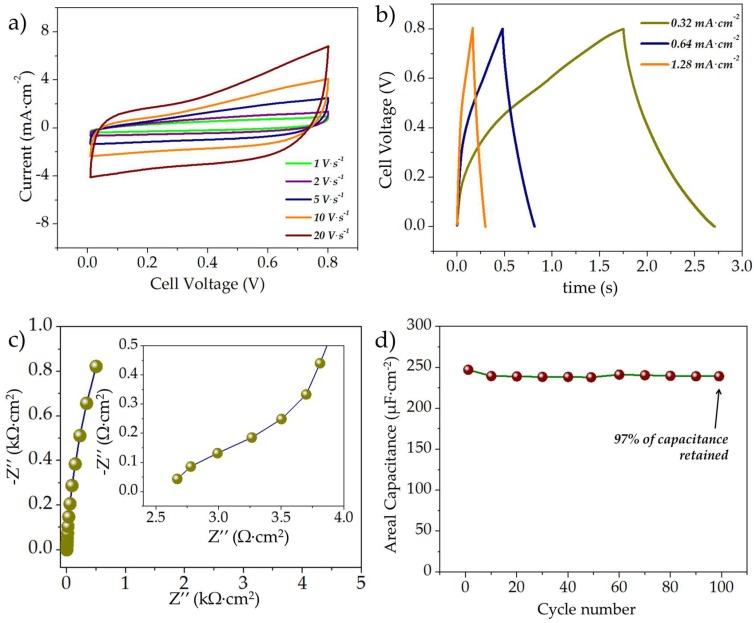
(**a**) Typical CV curves (scanned at 1–20 V·s^−1^); (**b**) GCD curves at different current densities; (**c**) EIS spectra (between 100,000–0.01 Hz) and (**d**) Cycle life of the symmetric rGO//rGO microsupercapacitor.

The electrochemical characteristics of the rGO//rGO symmetric MSC are shown in Figure 4. The typical CV curves of the symmetric rGO//rGO MSCs at scan rates of 1, 2, 5, 10, and 20 V·s^−1^ are shown in Figure 4a. As evident, even at a high scan rate of 20 V·s^−1^, the curves maintain a predominantly rectangular shape, which is indicative of capacitive charge storage. The typical GCD curves of the rGO//rGO MSCs at different current rates are shown in Figure 4b; the discharge areal capacitances were estimated as ~252, 223, and 172 µF·cm^−2^ at current densities of 0.32, 0.64, and 1.28 mA·cm^−2^, respectively. Figure 4c shows the typical Nyquist plots of the rGO//rGO MSCs scanned for a frequency range of 100,000–0.01 Hz; as evident the curves comprised a depressed semicircular region in the high frequency regime followed by a linear slope in the low-frequency region, indicating typical capacitive behavior; the diameter of the semicircular region was ~0.0035 kΩ·cm^2^ (~11 Ω), indicating very low charge transfer resistance; the x-axis intercept was ~8 Ω mainly due to solution resistance. The typical cycling behavior of the rGO//rGO microsupercapacitor is illustrated in Figure 4d; the capacitor exhibited very stable cycling with a capacitive retention of ~97% after 100 cycles.

#### 3.2.2. Electrochemical Characterization of the MnO_x_//MnO_x_ Symmetric MSCs

**Figure 5:**
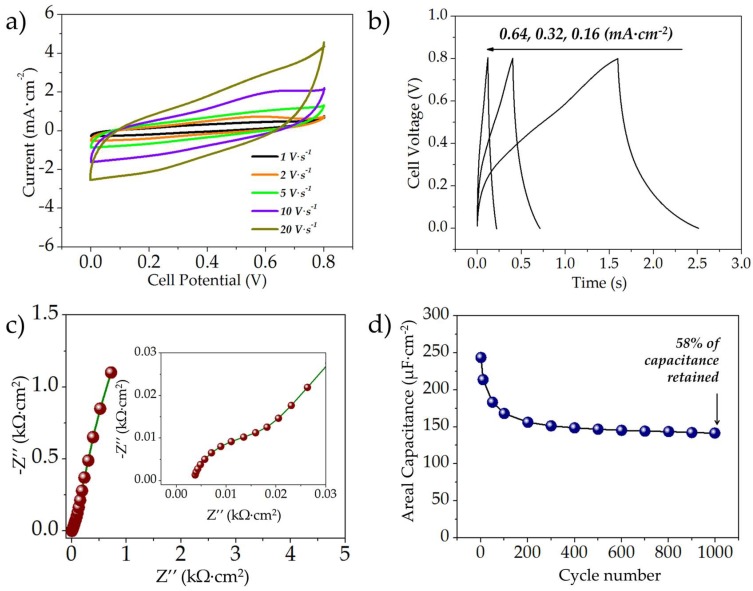
(**a**) Typical CV curves (scanned at 1–20 V·s^−1^); (**b**) GCD curves at different current densities; (**c**) EIS spectra (between 100,000–0.01 Hz) and (**d**) cycle life of the symmetric MnO_x_//MnO_x_ microsupercapacitor.

The electrochemical characteristics of the MnO_x_//MnO_x_ symmetric MSCs are shown in Figure 5. The typical cyclic voltammograms (CV) of the symmetric MnO_x_//MnO_x_ MSCs at scan rates of 1, 2, 5, 10, and 20 V·s^−1^ are shown in Figure 5a. As evident, even at a high scan rate of 20 V·s^−1^, the curves exhibit predominantly capacitive shape with some depressed redox peaks around 0.5–0.6 V, which could be attributed to the pseudocapacitive charge storage characteristics of the manganese oxide active material. The typical GCD curves at different current densities are shown in Figure 5b; the GCD curves display triangular sloping-desloping behavior for a voltage range of 0–0.8 V with some curvature. The discharge areal capacitances were estimated as 147, 96, 64 µF·cm^−2^ at current densities of 0.16, 0.32, 0.64 mA·cm^−2^, respectively. Figure 5c shows the typical Nyquist plots of the freshly prepared MnO_x_//MnO_x_ MSCs scanned for a frequency range of 100,000–0.01 Hz. As evident the curves comprised a depressed semicircular region in the high frequency regime followed by a linear slope in the low-frequency region, indicating typical redox behavior. The diameter of the semicircular region was ~0.02 kΩ·cm^−2^ (~60 Ω) indicating low charge-transfer resistance. The typical cyclability and the capacitance retention of the MnO_x_//MnO_x_ MSC are shown in Figure 5d; ~58% of the initial capacitance was retained after 1000 cycles. The drop in capacitance was observed in previous reports documenting the use of MnO_x_ for symmetric MSCs, and could be a result of the possible dissolution of the electro-active materials, which is an intrinsic issue with manganese oxides [43,44].

#### 3.2.3. Electrochemical Evaluation of the Asymmetric rGO//MnO_x_ MSCs

**Figure 6:**
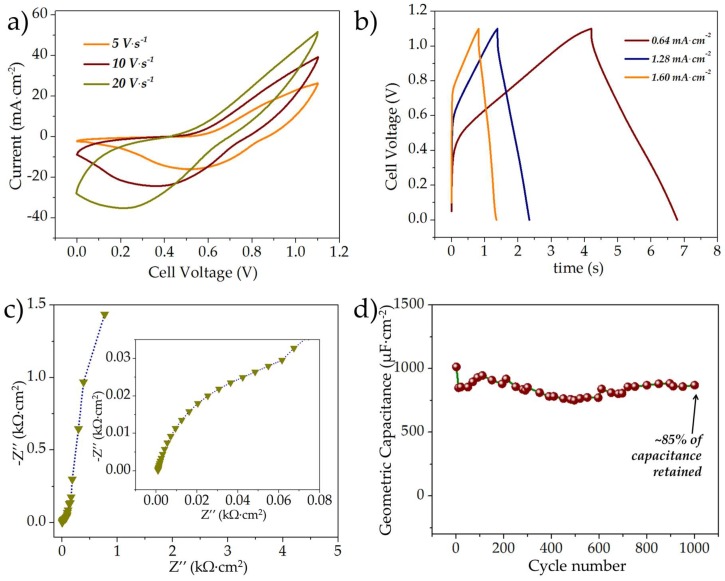
(**a**) Typical CV curves (scanned at 5–20 V·s^−1^); (**b**) GCD curves at different current densities; (**c**) EIS spectra (between 100,000–0.01 Hz) and (**d**) cycle life of the asymmetric rGO//MnO_x_-0.9C microsupercapacitor.

As seen in the previous sections, the areal capacitance of rGO//rGO MSC was higher than that of the MnO_x_//MnO_x_ MSC (~252 μF·cm^−2^ as opposed to 96 μF·cm^−2^ at a current density of 0.32 mA·cm^−2^), as a result of which, three different MnO_x_ thicknesses (MnO_x_ deposited at 0.6, 0.9, and 1.2 C·cm^−2^) were evaluated for asymmetric systems in order to investigate the optimal energy-power tradeoff. The asymmetric MSCs were designated as rGO//MnO_x_-0.6C, rGO//MnO_x_-0.9C and rGO//MnO_x_-1.2C, where the suffix symbolizes the charge used for MnO_x_ deposition. The electrochemical characteristics of the asymmetric rGO//MnO_x_-0.9C MSC are shown in Figure 6, whereas the electrochemical characteristics of the rGO//MnO_x_-0.6C and rGO//MnO_x_-1.2C MSCs can be found in the supplementary file (Figure S2). The typical CV curves of asymmetric rGO//MnO_x_-0.9C MSC at different scan rates are shown in Figure 6a. As evident the current response of the asymmetric rGO//MnO_x_-0.9C MSC was higher than that of both the symmetric rGO//rGO and MnO_x_//MnO_x_ capacitors. Furthermore, composite charge storage characteristics are evident from the shape of the CV curves. GCD curves of the asymmetric rGO//MnO_x_-0.9C MSC at different current densities are shown in Figure 6b; the discharge areal capacitances were estimated as 1.59, 1.29, and 1.2 mF·cm^−2^ at current densities of 0.64, 1.28, and 2.56 mA·cm^−2^, respectively. The Nyquist plot of the asymmetric rGO//MnO_x_-0.9C MSC scanned for a frequency range of 100,000–0.01 Hz is shown in Figure 6c and the inset depicts the zoomed-in high-frequency response of the MSC. The diameter of semicircular region was ~0.07 kΩ·cm^−2^ (~218 Ω), higher than both the symmetric rGO//rGO and MnO_x_//MnO_x_ MSCs. The relatively higher charge-transfer resistivity can be attributed to the larger thickness of the manganese oxide component on the asymmetric MSC. The typical cycling performance of the asymmetric rGO//MnO_x_-0.9C MSC is shown in Figure 6d; the capacitor retained a capacitance of ~85% after 1000 cycles, which is much superior to that of the MnO_x_//MnO_x_ MSC.

**Figure 7:**
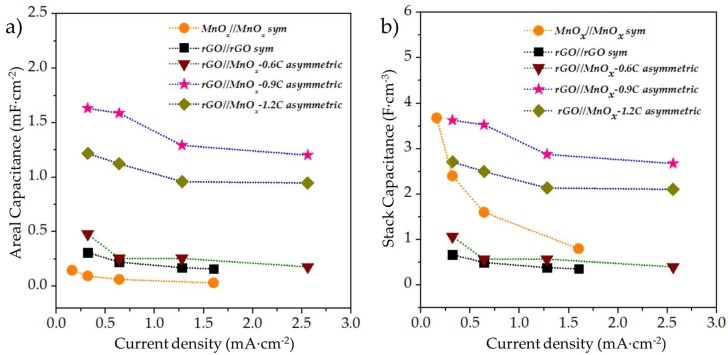
(**a**) Rate capability of the MnO_x_//MnO_x_, rGO//rGO, rGO//MnO_x_-0.6C, rGO//MnO_x_-0.9C, and rGO//MnO_x_-1.2C MSCs in terms of (**a**) areal and (**b**) stack capacitances.

The comparitive rate capability of the different MSCs evaluated in this work is shown in Figure 7a,b. While areal normalization is essential in terms of practicality of miniaturized devices, stack calculations can provide with more insight into the intrinsic properties of the materials utilized for the MSC construction. The stack capacitances of all the asymmetric MSCs as well as the rGO//rGO MSC were normalized with the thickness of the rGO film, whereas the stack parameters for the MnO_x_//MnO_x_ symmetric capacitor were normalized with the thickness of the MnO_x_ film. From Figure 7, it is clear that the asymmetric rGO//MnO_x_-0.6C, rGO//MnO_x_-0.9C, and rGO//MnO_x_-1.2C MSCs as well as the symmetric rGO//rGO MSC followed the same trend for both the areal and stack capacitances with the highest values for rGO//MnO_x_-0.9C followed by the rGO//MnO_x_-1.2C, rGO//MnO_x_-0.6C and the rGO//rGO MSC (in that order). The only discrepancy in stack and areal capacitance was exhibited by the symmetric MnO_x_//MnO_x_ system, which can be explained by the relatively lower thickness of the MnO_x_ as compared to the rGO film. The stack capacitance of the MnO_x_//MnO_x_ system fades quickly with increasing current, which can be attributed to the use of pristine MnO_x_ as electro-active material without the addition of any conducting additives.

**Figure 8:**
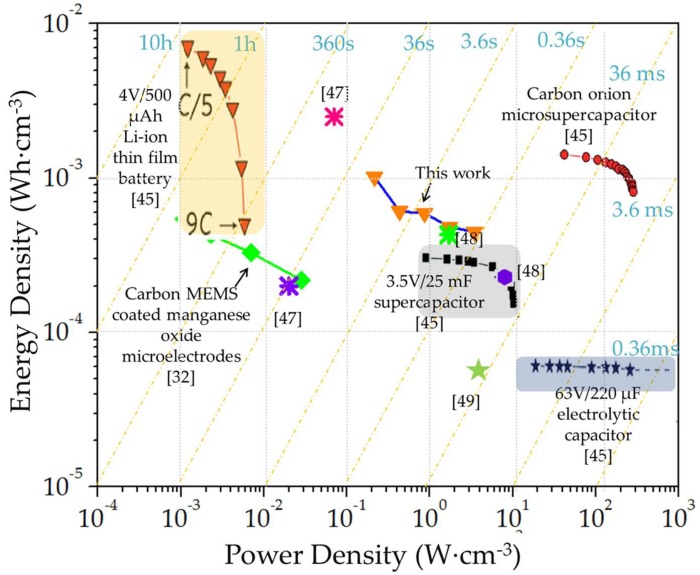
Ragone plot depicting the volumetric energy and power densities exhibited by the asymmetric rGO//MnO_x_-0.9C MSC in comparison with thin film lithium ion battery, commercial supercapacitors and electrolytic capacitors along with carbon onion-based microsupercapacitors (produced with permission from Reference [45]) and other data points taken from the Reference [32,47,48,49].

The areal capacitance, energy and power densities of the MSCs were computed using Equations (1)–(3), respectively

(1) $$C_{areal}=\frac{I\Delta t}{\Delta V}$$

(2) $$ED_{areal}=\frac{1}{2\times 3600}C_{areal}\times \Delta V^{2}$$

(3) $$PD_{areal}=\frac{ED_{areal}}{\Delta t/3600}$$

where *C_areal_* (F·cm^−2^), *ED_areal_* (Wh·cm^−2^) and *PD_areal_* (W·cm^−2^) refer to areal capacitance, energy and power density, respectively, *I* is the discharge current density (A·cm^−2^), Δ*V* is the voltage window (V), and Δ*t* is the discharge time (s). The stack capacitance, energy and power densities were calculated by normalizing the *C_areal_*, *ED_areal_* and *PD_areal_* with the rGO or MnO_x_ thickness, depending upon the higher thickness. The areal energy-power trade-off between all the MSC devices constructed in this work has been depicted in a Ragone chart (Figure S3); the asymmetric rGO//MnO_x_-0.9C MSC was able to deliver an energy density of 0.274 μWh·cm^−2^ at a power density of 193.6 μW·cm^−2^ (pertaining to a capacitance of 1.63 mF·cm^−2^) which is higher than other reports documenting on-chip MnO_x_-based systems [32,35] but lower than reports on carbon onions and conjugated polymers [45,46]. A comparative Ragone chart depicting the stack energy and power densities of the asymmetric rGO//MnO_x_-0.9C MSC along with other commercial EES [45] as well as other MSC systems reported in the literature is shown in Figure 8. Areal and stack characteristics of the asymmetric MSC have also been compared with other on-chip MSCs in Table 1. The asymmetric MSC was able to deliver a maximal stack energy density of 1.02 mWh·cm^−3^ at a power density of 0.22 W·cm^−3^, and a maximal power density of 3.44 W·cm^−3^ at which an energy density of 0.45 mWh·cm^−3^ was maintained, resulting in a time constant of 0.47 s. It can be seen that the hybrid MSC is well-placed in between the energy-power characteristics of thin film lithium-ion battery and commercial supercapacitors. It should be noted that the device characteristics have been reported in terms of volume of the interdigitated fingers and the values will diminish taking packaging into account. Furthermore, the coulombic efficiency of the MSC systems was relatively low, which could be ascribed to the predominant use of oxides and improvement is expected with the addition of nanostructured conducting agents. Additionally, while the aqueous asymmetric MSC displays high energy and power tradeoff, further enhancement in electrochemical performance is expected with the use of high voltage electrolytes, advanced hierarchical materials, as well as more balanced hybrid designs, which are subjects of future works.

## 4. Conclusions

In summary, interdigitated microsupercapacitors were fabricated using photolithography, lift-off and electrodeposition methods. Symmetric MnO_x_//MnO_x_, rGO//rGO as well as asymmetric rGO//MnO_x_ microsupercapacitors with three different MnO_x_ thicknesses were constructed and evaluated. The asymmetric microsupercapacitor with the MnO_x_ film deposited at a charge of 0.9 C·cm^−2^ displayed the optimal energy-power trade-off much superior to that of both the symmetric and well as the other asymmetric configurations. The rGO//MnO_x_-0.9C microsupercapacitor exhibited a high stack energy density of 1.02 mWh·cm^−3^ and a maximal power density of 3.44 W·cm^−3^ both of which are comparable with thin film batteries and commercial supercapacitors in terms of volumetric energy and power densities, respectively. The high energy-power trade-off of the device is attributed to the synergistic effects of utilizing double layer and pseudocapacitive charge storage mechanisms along with in-plane interdigital design within one optimized micro-device.

## Figures and Tables

**Table 1:** Comparison of the asymmetric rGO//MnO_x_ MSC with on-chip MSCs reported in the literature.

Device Design	Electro-Active Materials	Electrolyte	Specific Capacitance	Energy-Power Characteristics	Ref
**Sandwich**	Carbon Microelectromechanical systems (C-MEMS) coated manganese oxide	Aqueous 1 M Na_2_SO_4_	Maximal areal capacitance of 0.055 F·cm^−2^ and stack capacitance of 7.4 F·cm^−3^	Stack energy and power densities of 0.51 mWh·cm^−3^ and 28.3 mW·cm^−3^, respectively	[32]
**Interdigital**	Graphene quantum dots//manganese oxide	Aqueous 0.5 M Na_2_SO_4_	1.1 mF·cm^−2^	0.154 μWh·cm^−2^ at a specific power of 7.51 μW·cm^−2^	[36]
**Interdigital**	Onion-like carbon	1 M Et_4_NBF_4_ in PC	1.35 F·cm^−3^ at 1 V·s^−1^	Stack energy density of ~1.7 mWh·cm^−3^ and power density of 200–250 W·cm^−3^	[45]
**Interdigital**	Nano-porous gold/MnO_2_	(PVA)/H_2_SO_4_	-	Energy density of 55 μWh·cm^−3^ Power density of 3.4 W·cm^−3^	[49]
**Interdigital**	Manganese oxide Reduced graphene oxide	1 M Na_2_SO_4_	Maximal areal capacitance of 1.63 mF·cm^−2^, equivalent to a stack/volumetric capacitance of 3.6 F·cm^−3^	Maximal energy density of 1.02 mWh·cm^−3^ Maximal power density of 3.44 W·cm^−3^ Areal energy density of 0.274 μWh·cm^−2^ at a power density of 193.6 μW·cm^−2^	This work

## Supplementary Materials

The following are available online at http://www.mdpi.com/2072-666X/9/8/399/s1, Figure S1: Microstructure of the single layer graphene oxide (SLGO); Figure S2: Typical cyclic voltammograms at different scan rates of (a) the asymmetric rGO//MnOx-0.6C MSC and (b) the asymmetric rGO//MnOx-1.2C MSC; typical GCD curves of (c) the asymmetric rGO//MnOx-0.6C MSC and (d) the asymmetric rGO//MnOx-1.2C MSC; cycle life of (e) the asymmetric rGO//MnOx-0.6C MSC and (f) the asymmetric rGO//MnOx-1.2C MSC; Figure S3: Ragone chart of the different MSC systems.
