# Supplementary material for: On-Chip Asymmetric Microsupercapacitors Combining Reduced Graphene Oxide and Manganese Oxide for High Energy-Power Tradeoff
Source: Micromachines (Basel). 2018 Aug 12;9(8):399. doi: 10.3390/mi9080399 (PMC6187318; doi:10.3390/mi9080399)
Supplement: Supplementary file 1 [file micromachines-09-00399-s001.pdf]

Supplemental Information

On-Chip Asymmetric Microsupercapacitors Combining Reduced Graphene Oxide and Manganese Oxide for High Energy-Power Tradeoff

Richa Agrawal[1] *and Chunlei Wang*[1,2 ‡]

[1] Department of Mechanical and Materials Engineering, Florida International University, Miami, FL 33174, United States

[2] Center for the Study of Matter of Extreme Conditions (CeSMEC) Florida International University, Miami, FL 33199, United States

‡ Author to whom correspondence should be addressed: wangc@fiu.edu

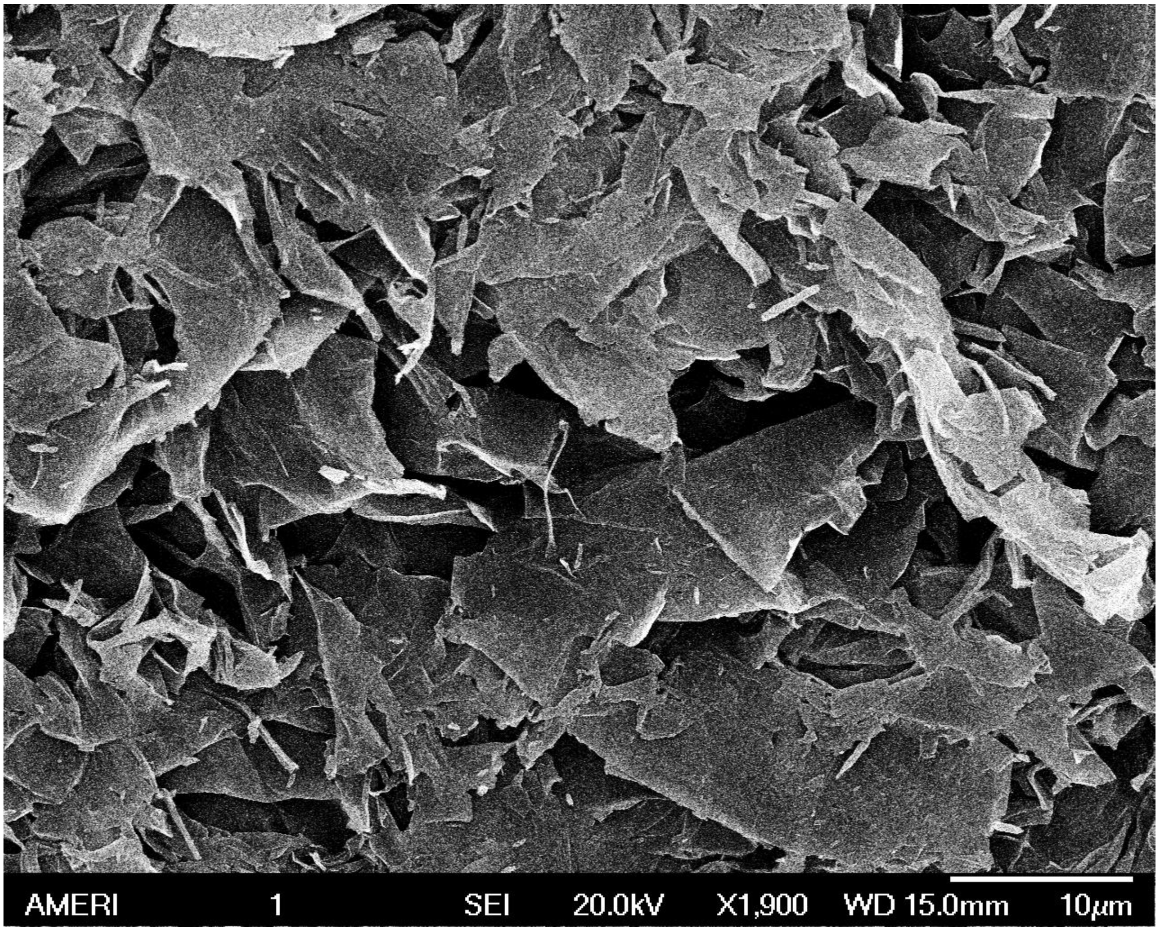

Figure S1: Microstructure of the single layer graphene oxide (SLGO)

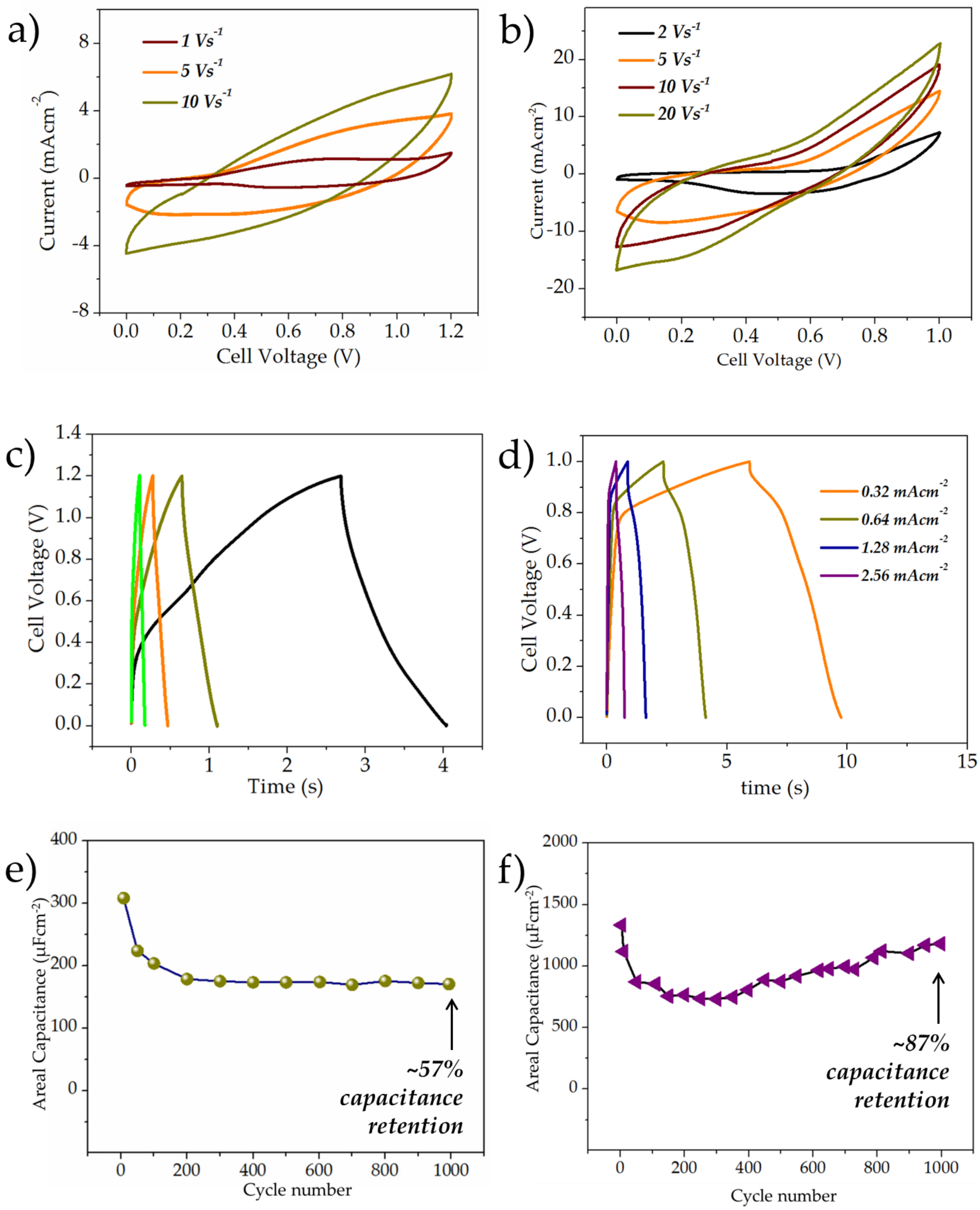

Figure S2: Typical cyclic voltammograms at different scan rates of a) the asymmetric rGO//MnO_x-0.6C MSC and b) the asymmetric rGO//MnO_x-1.2C MSC; typical GCD curves of c) the asymmetric rGO//MnO_x-0.6C MSC and d) the asymmetric rGO//MnO_x-1.2C MSC; cycle life of e) the asymmetric rGO//MnO_x-0.6C MSC and f) the asymmetric rGO//MnO_x-1.2C MSC.

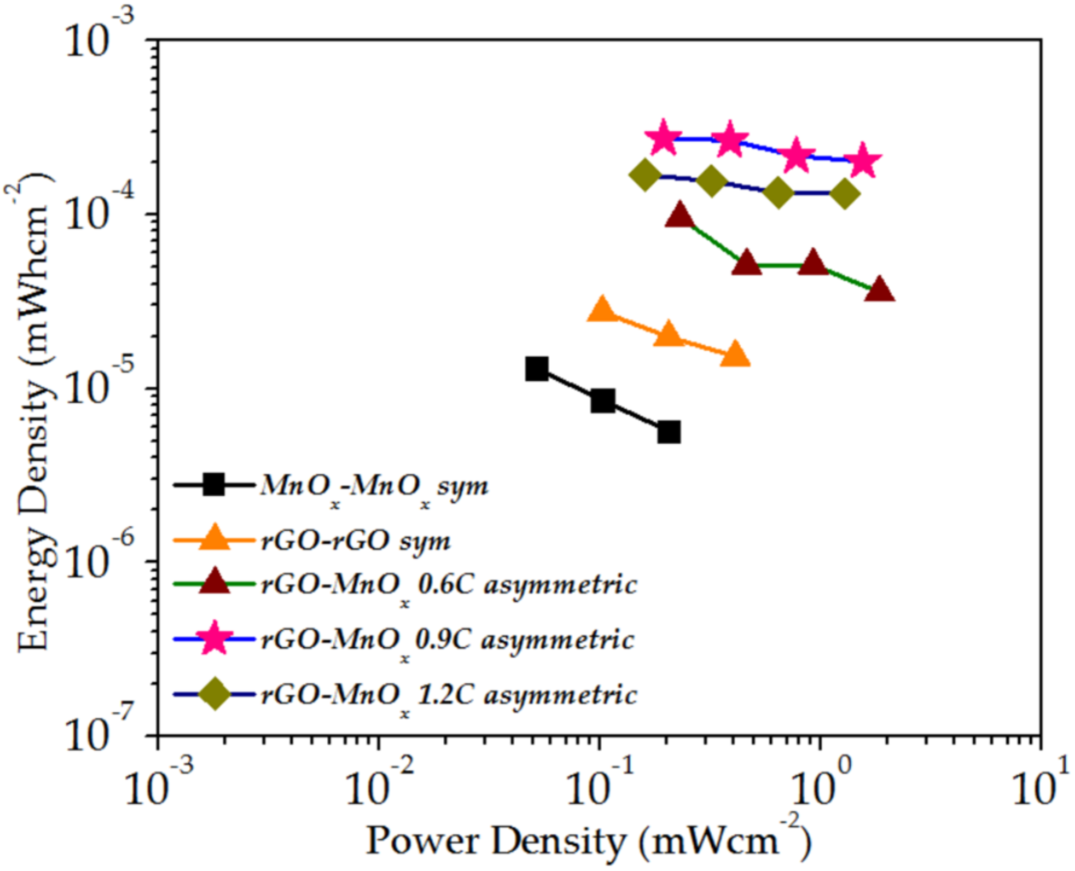

Figure S3: Ragone chart of the different MSC systems.
